# Cross-Linked Gel Polymer Electrolyte Based on Multiple Epoxy Groups Enabling Conductivity and High Performance of Li-Ion Batteries

**DOI:** 10.3390/gels9050384

**Published:** 2023-05-05

**Authors:** Wei Zhang, Wansu Bae, Lei Jin, Sungjun Park, Minhyuk Jeon, Whangi Kim, Hohyoun Jang

**Affiliations:** Department of Applied Chemistry, Konkuk University, Chungju-si 27478, Republic of Korea

**Keywords:** cross-linked gel polymer electrolyte, LiFSI, ionic conductivity, interfacial stability, Coulombic efficiency

## Abstract

The low ionic conductivity and unstable interface of electrolytes/electrodes are the key issues hindering the application progress of lithium-ion batteries (LiBs). In this work, a cross-linked gel polymer electrolyte (C-GPE) based on epoxidized soybean oil (ESO) was synthesized by in situ thermal polymerization using lithium bis(fluorosulfonyl)imide (LiFSI) as an initiator. Ethylene carbonate/diethylene carbonate (EC/DEC) was beneficial for the distribution of the as-prepared C-GPE on the anode surface and the dissociation ability of LiFSI. The resulting C-GPE-2 exhibited a wide electrochemical window (of up to 5.19 V vs. Li^+^/Li), an ionic conductivity (σ) of 0.23 × 10^−3^ S/cm at 30 °C, a super-low glass transition temperature (T_g_), and good interfacial stability between the electrodes and electrolyte. The battery performance of the as-prepared C-GPE-2 based on a graphite/LiFePO_4_ cell showed a high specific capacity of ca. 161.3 mAh/g (an initial Coulombic efficiency (CE) of ca. 98.4%) with a capacity retention rate of ca. 98.5% after 50 cycles at 0.1 C and an average CE of about ca. 98.04% at an operating voltage range of 2.0~4.2 V. This work provides a reference for designing cross-linking gel polymer electrolytes with high ionic conductivity, facilitating the practical application of high-performance LiBs.

## 1. Introduction

LiBs have been extensively used in consumer electronic and storage devices, owing to their high specific energy density, stable cycling performance, high open circuit potentials, low memory effects, light weight, and so forth [1,2,3]. Electrolytes play a critical role in high-performance LiBs. However, commercial liquid electrolytes (LEs) generate lithium dendrite, an unstable solid electrolyte interface (SEI), and leakage and flammability of the LE [4,5,6]. Polymer electrolytes have been extensively studied to solve this problem due to their significant advantages of low flammability, excellent processability, high electrochemical stability, good mechanical flexibility, and durability [7]. Gel polymer electrolytes (GPEs), which incorporate liquid plasticizers and lithium salts into polymer matrices, have been identified as one of the most promising candidates for cost-effective, safe, and long-lifespan LiBs [6,8]. GPEs are nonvolatile, have higher thermal and electrochemical stability than LEs, and, to some extent, alleviate problems derived from water [4,9]. Moreover, GPEs can be manufactured using the in situ polymerization method, which is compatible with the state-of-the-art LiB fabrication industry [10,11,12]. Among in situ-formed GPEs, polyethylene oxide (PEO), polymethyl methacrylate (PMMA), polyvinylidene fluoride (PVDF) [13,14], polyacrylonitrile (PAN) [10], poly (1,3-dioxlane) [15], and related co-block polymers are currently widely used as polymer hosts for the preparation of GPEs. Nevertheless, the ionic conductivity (σ) and battery performance of PEO-based GPEs still need to be improved, owing to the crystallinity of the as-prepared electrolyte and interfacial issues. For example, Xie et al. [16] present a dual-salt PEO-based cross-linked network electrolyte with a σ value of 0.57 × 10^−3^ S/cm at 30 °C, while Teng et al. [9] present a poly(ethylene oxide)-co-poly(propylene oxide)-based GPE that delivers a σ value of 2.8 × 10^−3^ S/cm at 30 °C, but polarization occurs due to anion accumulation. Our group’s recent work employed LiFSI as an initiator and successfully polymerized a dual-epoxy group precursor through thermal cationic ring-opening technology [6,17,18]. The results showed tight interface compatibility between the electrolyte and electrode, which enables the σ and cycling performance of the battery.

Apart from LEs, GPEs with a certain amount of solvent might facilitate battery performance. For example, Lai et al. [19] report an in situ-formed GPE with 1 M LiPF_6_ in EC/DEC/EMC that has a more stable deposition and dissolution behavior due to the uniform Li cation distribution on the anode surface. Wang et al. [20] present an in situ-formed solid-state polymer electrolyte based on poly(1,3-dioxolane) with a high σ (7.9 × 10^−3^ S/cm at room temperature), a high Li cation transference number (0.82), and low interfacial resistance. However, employing highly concentrated LiFSI (3.5 M) probably sacrifices cost-effectiveness and higher polymer electrolyte molecular weights due to rapid polymerization. Thus, a moderate condition needs to be considered. Furthermore, for comparison, LiTFSI-/LiPF_6_-based GPEs [5,21,22,23] and LiFSI-based GPEs have the merits of higher σ of LiFSI [24,25], better interface compatibility with the electrodes, and better cycling performance due to the formation of the cathode–electrolyte interphase (CEI) [26] and solid–electrolyte interphase (SEI) layer [20] on the anode surface. From another aspect, the PEO-based SPEs face the challenges of poor ionic conductivity and interface issues by adding a plasticizer (e.g., fluoroethylene carbonate and succinonitrile) [15,27]. In contrast, cross-linked PEO-based GPEs could possess thermal ability, improved σ, and enhanced interfacial stability [14,28].

Epoxidized soybean oil (ESO), as a renewable raw material with multiple epoxy groups, has attracted great attention in recent years in both scientific and industrial areas, such as in fabricating lubricants, coatings, and bioplastics [29,30,31]. Research has explored the ring-opening polymerization of ESOs through boron trifluoride diethyl etherate (BF_3_.OEt_2_), and the epoxy group-based precursor can successfully achieve cationic ring-opening polymerization using Li salts, including LiFSI, LiPF_6_, and lithium difluoro (oxalate) borate (LiDFOB) [4,17,22].

Herein, we attempted to prepare an in situ cross-linking gel polymer electrolyte (C-GPE), which consisted of low-concentrated LIFSI, environmentally friendly ESO, and EC/DEC solvent (1:1, by volume). The EC/DEC solvent could be beneficial for the dissociation of LiFSI and adjust the interfacial stability of the electrolyte and anode [32,33], while the ESO-based electrolyte probably provides a three-dimensional (3D) cross-linked structure, which improves ionic conductivity and provides more volume for ion mobility. In situ polymerization could also enhance battery performance by reducing interfacial resistance and generating intimate compatibility between the electrolyte and electrode [6,10,11,12,13]. In this work, we expect to provide a simple and practical method for designing C-GPEs with outstanding ionic conductivity, a wide electrochemical stability window, and low interfacial impedance for high-performance rechargeable LiBs.

## 2. Results and Discussion

### 2.1. Ionic Conductivity and Electrochemical Properties

**Figure 1:**
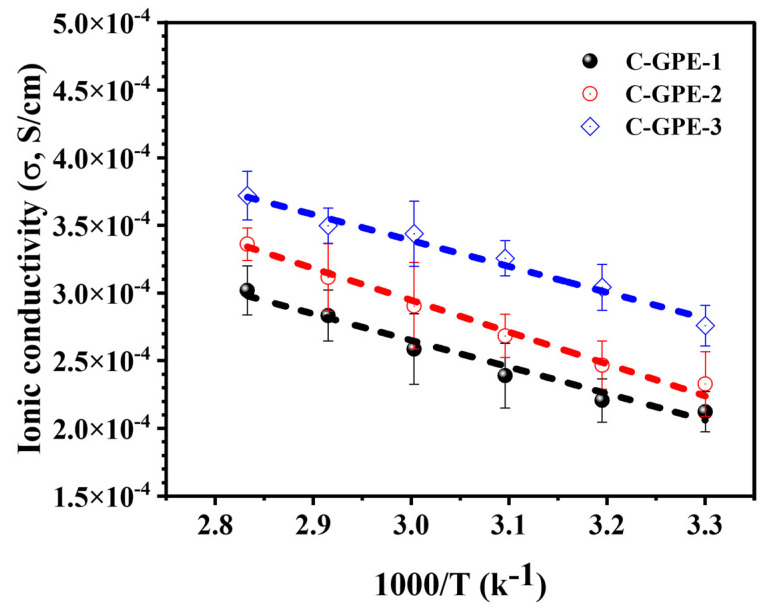
Plots of ionic conductivity vs. temperature based on dummy cells.

As can be seen in Figure 1, the ionic conductivities of the as-synthesized C-GPEs are measured using Figure S1a–d of the Supplementary Materials (SM) and Equation (1), whose values are comparable to those of the reported GPEs. All as-prepared C-GPEs delivered a considerable σ (compared with Table S1 of the SM). At 30 °C, the typical σ values of the as-prepared C-GPEs—C-GPE-1, C-GPE-2, and C-GPE-3—were 0.21, 0.23, and 0.28 × 10^−3^ S/cm, respectively, while at 80 °C, the values of the corresponding σ were 0.3, 0.33, and 0.37 × 10^−3^ S/cm, respectively. Furthermore, a linear dependency of ln σ along with the temperature was displayed, which agrees with the typical Arrhenius plot [18]. Combined with battery performance, as shown below, these comparable σ values may be due to the increased distribution of free Li cations in the interface region [13], suggesting that EC/DEC facilitates the dissolution of Li ions, thereby improving the σ value.

**Figure 2:**
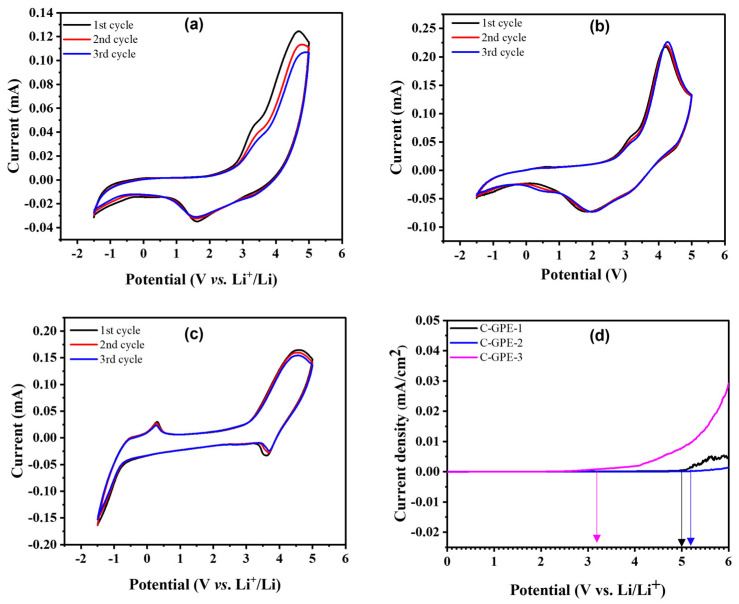
CV profiles of as-prepared (**a**) C-GPE-1, (**b**) C-GPE-2, and (**c**) C-GPE-3; (**d**) LSV traces of as-prepared C-GPEs based on Li//SS cells with a potential of 0–6 V and a scan rate of 0.1 mV/s.

Figure 2a–c and Figure S2 of the SM display the CV profiles of the as-prepared C-GPEs based on half-cells (Li/C-GPEs/LiFePO_4_) and all clearly exhibit redox peaks. The CV peaks from C-GPE-1 and C-GPE-2 were found at 4.5 and 1.6 (Figure 2a) and 4.3 and 1.8 V (Figure 2b), respectively. These observations could indicate the de-lithiation and lithiation of the electrodes. Furthermore, the CV curves of C-GPE-2 overlap for three cycles, indicating the normal reversibility of the electrochemical reaction within the voltage range of −1.5~5 V. However, Figure 2c and Figure S2 (C-GPE-4) of the SM show an extra peak at 0.2 V, which may be attributed to a side reaction due to the higher fraction of solvent (EC/DEC). Electrochemical anodic stability is a critical parameter of LiBs. As Figure 2d shows, the stable window values of the as-prepared C-GPEs based on Li||SS cells for C-GPE-1, C-GPE-2, and C-GPE-3 were 5.0, 5.19, and 3.2 V, respectively. The as-prepared C-GPE-2-based cell exhibited a wide and stable voltage, which may be attributed to the good distribution of LiFSI and less to the solvation effect of EC/DEC [34,35].

### 2.2. Battery Performances

**Figure 3:**
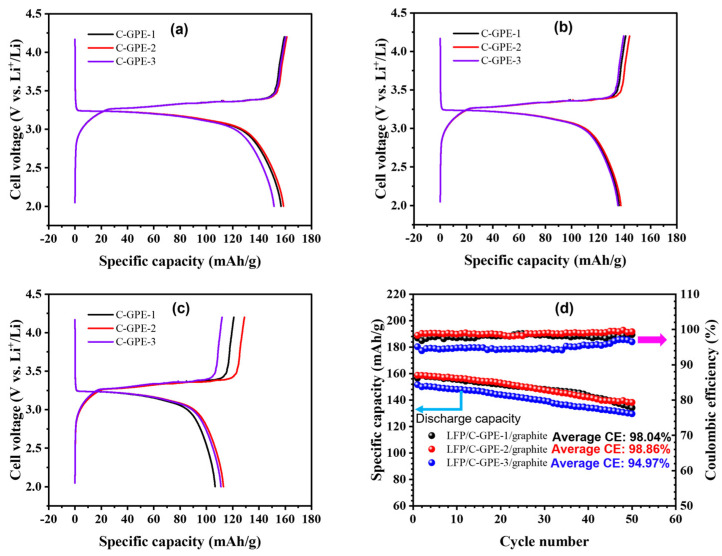
CD plots of graphite/C-GPEs/LFP cells at 0.1 C (**a**), 0.2 C (**b**), and 0.3 C (**c**) under 25 °C. (**d**) Plots of discharge-specific capacity as a function of CD cycles at 25 °C for as-prepared C-GPE-based graphite/C-GPEs/LFP full cells at 0.1 C.

The rate performances of the as-prepared C-GPEs were tested using a full-cell configuration of graphite/C-GPEs/LiFePO_4_ (LFP) with a potential range of 2.0–4.2 V at 0.1 C, 0.2 C, and 0.3 C, respectively. Figure 3a–c display variations in the discharge–charge (CD) capacity at 0.1–0.3 C. At 0.1 C, the initial charge- and discharge-specific capacities (C_sp_) of C-GPE-1 were ca. 159.4 and 156.8 mAh/g, with an initial Coulombic efficiency (CE, η) of ca. 99.6%. Meanwhile, C-GPE-2 showed ca. 161.3 and 158.7 mAh/g with an η of ca. 98.4%, while C-GPE-3 showed ca. 160.3 and 151.5 mAh/g with an η of 94.5%, respectively. Furthermore, at 0.2 C, the C_sp_ of C-GPE-1, C-GPE-2, and C-GPE-3 were ca. 136.1, 137.5, and 135.2 mAh/g, respectively. At 0.3 C, the C_sp_ of C-GPE-1, C-GPE-2, and C-GPE-3 were ca. 106.7, 113.0, and 111.1 mAh/g, respectively. These good rate capacities of the as-prepared C-GPEs based on a full cell at 0.1–0.3 C could be attributed to the better interfacial compatibility between the cathode and electrolyte [36,37]. These observations are higher than or comparable with recent reported works (as shown in Table S1 of the SM). As can be seen in Figure 3d, the average η of the graphite/C-GPE-2/LFP battery is ca. 98.86%; nevertheless, the values of the C-GPE-1- and C-GPE-3-based full cells are ca. 98.04 and 91.97%, respectively, at 0.1 C after 50 CD cycles. The lower average η of the C-GPE-3 battery cell might be attributed to an unstable interface between the anode/C-GPE-3 (verified by Figure 2c). Furthermore, this result could be due to the solvation effect of Li ions [38]. It is worth noting that the slightly higher average η (98.86%) of the C-GPE-2 battery cell than that (98.04%) of the C-GPE-1 battery cell is probably due to the formation of a stable solid electrolyte layer on the graphite in the presence of LiFSI [6] and the suitable amount of EC/DEC adjusting the interfacial behavior on the graphite anode surface [32] with the increasing cycle number.

**Figure 4:**
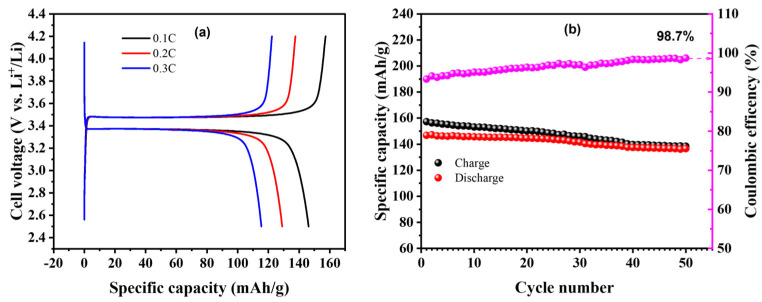
(**a**) Rate performances of Li/C-GPE-2/LFP half-cell at 0.1–0.3 C and 25 °C. (**b**) Discharge–charge capacities and CEs for Li/C-GPE-2/LFP half-cell at 0.1 C for 50 cycles at 25 °C.

Encouraged by the considerable performance in full-cell configuration (graphite/C-GPE-2/LFP), a half-cell (Li/C-GPE-2/LFP) was assembled to demonstrate its potential for large-scale applications. As shown in Figure 4a,b, the rate and cycling performance of the as-prepared C-GPE-2-based half-cell (Li/C-GPE-2/LFP) were investigated. The C_sp_ of the as-prepared C-GPE-2-based half-cell at 0.1, 0.2, and 0.3 C was ca. 146, 129.0, and 115.4 mAh/g, respectively. At the same time, there is less polarization potential (as displayed in Figure 4a). These findings indicate that C-GPE-2 may have good compatibility with Li metal anodes [32,33,39]. Figure 4b shows the C_sp_ and cyclability of the as-prepared C-GPE-2-based half-cell with 50 cycles at 2.5–4.2 V and 25 °C. This makes it clear that the slightly increasing value of η (up to 98.7%) could infer the generation of uniform Li^+^ and a stabler SEI layer on the metallic Li anode surface. Compared to the initial C_sp_ (ca. 146.8 mAh/g) with an η of ca. 93.32%, the capacity retention of the Li/C-GPE-2/LFP battery was maintained at ca. 136.6 mAh/g (ca. 93.05%) after 50 cycles at 0.1 C and 25 °C.

### 2.3. Morphological Characterization

**Figure 5:**
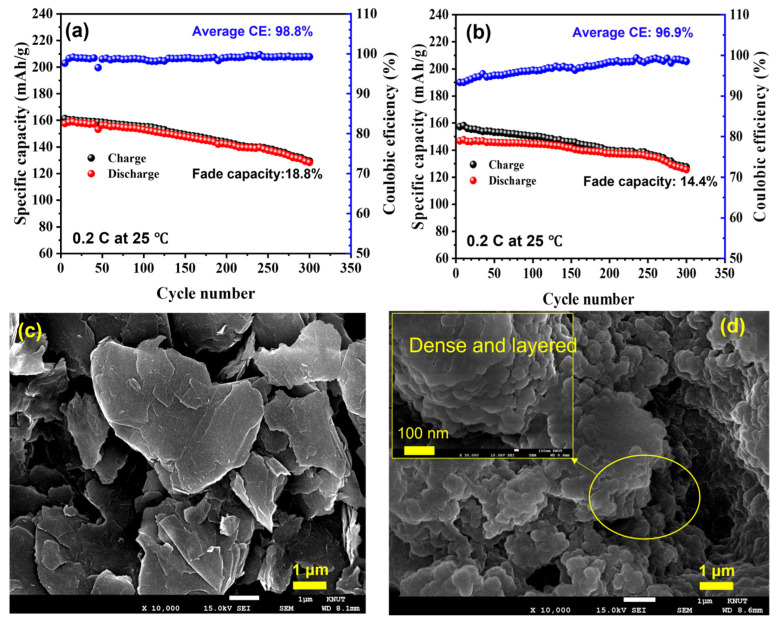
Variation in the specific capacity and the Coulombic efficiency as a function of CD cycling number of the corresponding full cell (**a**) and half-cell (**b**) at 0.2 C and 25 °C; FE-SEM images of (**c**) pristine graphite anode and (**d**) graphite anode based on C-GPE-2 electrolyte-contained LiBs (inset image shows the corresponding magnified FE-SEM image).

Figure 5a,b show the variations in the specific capacities and CEs vs. the CD cycling number of the as-prepared C-GPE-2 based on a full cell and a half-cell, respectively, at 0.2 C and 25 °C. The fade C_sp_ for the as-prepared C-GPE-2 based on the cells was ca. 18.8 and 14.4%, respectively, after 300 cycles. The corresponding average CE for the as-prepared C-GPE-2-based LiBs was ca. 98.8 and 96.9% after cycling. As can be seen, the monotonic increase in CE with the increasing CD cycling number can be attributed to the formation of an SEI layer on the graphite anode induced by the electrolyte [6]. Nevertheless, the average CE of the as-prepared C-GPE-2 electrolyte-based full cell is higher than that of the corresponding half-cell. This is possibly due to the better interface compatibility of graphite anodes compared to Li metal anodes. The full-cell interface stability of the LiBs based on C-GPE-2 was further investigated by analyzing the surface morphologies of the graphite anode. Figure 5c displays the FE-SEM image of the pristine graphite anode, and Figure 5d shows the FE-SEM image of the graphite anode of the as-prepared C-GPE-2-based full cell. After 300 CD cycles of the as-prepared C-GPE-2-based full cell, the graphite was recovered. It revealed that the as-prepared C-GPE-2 is capable of producing a dense, layered, and stable SEI layer on the graphite anode. This SEI layer can extend the interface stability of the electrolyte and electrode, which concurrently enhances the lifespan of LiBs [40]. The formation of the SEI layer was identified further by measuring the EDS elemental mapping of the graphite anode, as shown in Figure S5. The EDS elemental mapping clearly indicates the existence of a high density of the elements C, O, F, S, and N in the graphite anode.

### 2.4. Characterization of As-Synthesized C-GPEs

**Figure 6:**
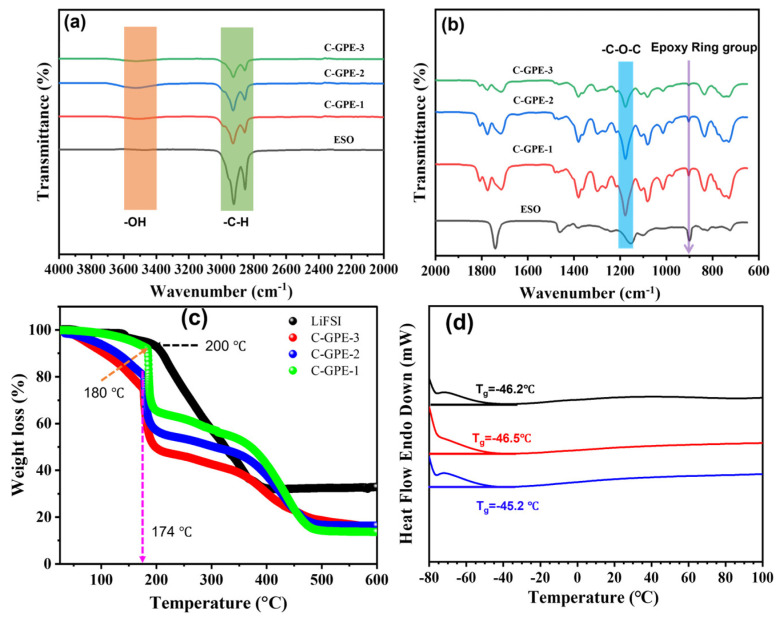
(**a**,**b**) FTIR spectra of as-prepared C-GPEs and ESO; (**c**) TGA traces of as-synthesized C-GPEs and LiFSI salt; (**d**) DSC plots of as-prepared C-GPEs.

Figure 6a,b show the FTIR spectra that were obtained to gain further insight into the structural characterization of the as-prepared C-GPEs. After completing polymerization for 24 h, all of the as-prepared C-GPEs display a broad peak (ca. 3505 cm^−1^) of hydroxyl O–H stretching and reduced intensity of epoxy ring group stretching (ca. 902 cm^−1^), suggesting that the ring-opening process was conducted. The peak at ca. 826 cm^−1^ is assigned to epoxy group stretching. These results are in good agreement with previously reported work [18,29]. Additionally, the stretching of C–H was found at a peak of ca. 2840–2946 cm^−1^, and the peaks at ca. 1700–1800 cm^−1^ and ca. 1178 cm^−1^ of the as-prepared C-GPEs are attributed to the C=O stretching from EC/DEC [8] and the C–O–C deformation vibration [31,41], respectively.

Figure 6c shows the thermogravimetric analysis results of the LiFSI and the as-prepared C-GPEs. Obviously, the relative stable temperatures of the as-prepared C-GPE-2 and C-GPE-3 (up to ca. 174 °C) were similar, whereas the as-prepared C-GPE-1 (up to ca. 180 °C) exhibited a lower mass loss, which could mainly be attributed to moisture and the solvent (EC/DEC). After that, the degradation of the as-prepared C-GPEs was taken at a temperature range of ca. 180–480 °C. In contrast, the LiFSI had already decomposed at ca. 142 °C [42]. The enhanced thermal stability can be attributed to the conversion degree of the polymer and the cross-linked structure [20], which is also verified by the FTIR and DSC results. DSC measurements were conducted after the as-prepared C-GPEs were dried in a vacuum oven for 12h at 80 °C. As can be seen in Figure 6d, the glass transition temperatures (T_g_) of the as-prepared C-GPEs—C-GPE-1, C-GPE-2, and C-GPE-3—were ca. −45.2, −46.5, and −46.2 °C, respectively. The super-low T_g_ values of the as-prepared C-GPEs are significantly linked to their highly amorphous network [41], which is confirmed by the XRD patterns.

### 2.5. In Situ Cross-Linking Gel Polymer Electrolytes

**Figure 7:**
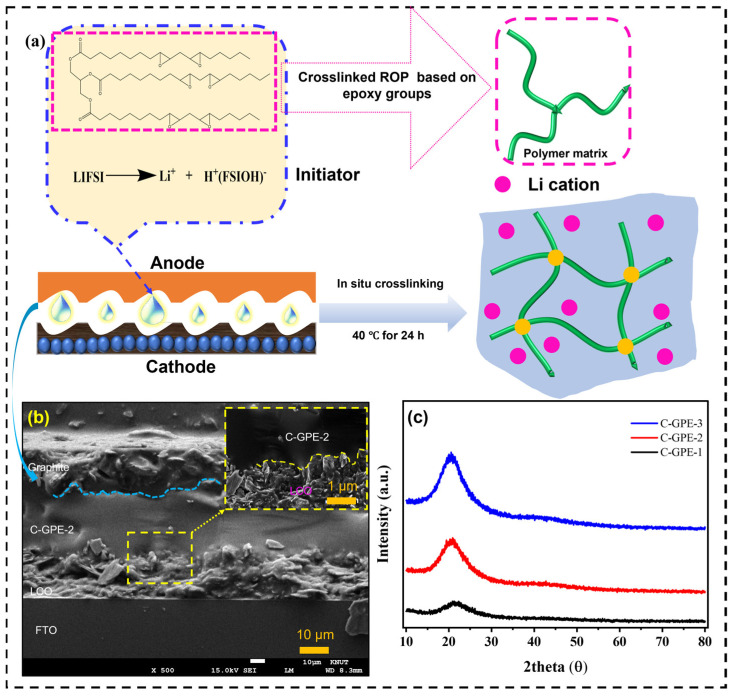
(**a**) Schematic diagram of in situ-fabricated C-GPEs. (**b**) Cross-sectional FE-SEM image of as-prepared C-GPE-2-based dummy cell (the inset is the enlarged area between the LCO cathode/the C-GPE-2). (**c**) XRD plots of as-prepared C-GPEs.

Figure 7a shows a plausible in situ cross-linking process and the proposed evolutionary network. Generally, in the first step, the homogeneous precursor solution containing the LiFSI, monomer (ESO), and solvent (EC/DEC) was injected into the assembled battery. Next, LiFSI provides Lewis acid H (FSIOH) under the heating condition of 40 °C [6], which attacks the cyclic epoxide ring and continues the propagation and cross-linking cationic ring-opening polymerization in the ESO chain in the presence of EC/DEC. Finally, the 3D cross-linked polymer network in the C-GPEs is formed in situ, directly inside the battery. Notably, this fabrication process is compatible with the current industrial application of LiBs. As additional initiators (e.g., LiTFSI and LiBF_4_) [4,15,43,44] and cost-ineffective additives/plasticizers (e.g., FEC) [15] are unnecessary, this as-prepared C-GPE offers similar, or even lower, expense than the other reported works [45]. Figure 7b shows cross-sectional images investigating the close interface affinity between the cathode/as-prepared C-GPE-2/graphite anode, respectively. This tight contact between the cathode and the as-prepared electrolyte enables Li-ion communication and facilitates cycling performance [15,36,37]. Moreover, XRD tests were utilized to measure the crystallization of the as-prepared C-GPEs. As depicted in Figure 7c, all of the as-prepared C-GPEs showed a broad peak at around 2θ = 21.6°, suggesting a high-degree amorphous morphology with chain mobility [6,15]. The XRD analysis matches well with the T_g_ results of the as-prepared C-GPEs. Notably, the enhanced intensity of the amorphous peak should be due to the addition of EC/DEC, which enables the mobility of the polymer chain, as well as the increased FHMW.

## 3. Conclusions

In summary, we report an in situ-formed C-GPE based on poly (ESO), which is successfully developed to simultaneously improve the interface adhesion and generate uniform distribution on an anode surface in the presence of 20% (*v*/*v*) solvent (EC/DEC). The as-synthesized C-GPEs exhibited good thermal stability (stable up to 180 °C), super-low T_g_ values, and considerable electrochemical properties. The FE-SEM images revealed a tight contact between the electrolyte and electrodes, which facilitates battery performance. Especially, the as-synthesized C-GPE-2 showed a T_g_ value of −46.5 °C, a comparable σ at 30 °C, and a broader electrochemical stability window (up to 5.19 V). As a result, the graphite/C-GPE-2/LFP full cell delivered an initial C_sp_ of ca. 158.7 mAh/g with an average η of 98.86% after 50 CD cycles at 0.1 C. Moreover, the as-prepared C-GPE-2-based Li//LFP half-cell delivered a capacity retention of ca. 93.05% after 50 CD cycles at 0.1 C. Additionally, the capacity retention of the as-prepared C-GPE-2-based cells was over 80% after 300 CD cycles. These results could provide an effective approach to improving anode stability and adjusting the distribution of electrolyte on electrode surfaces in lithium gel polymer batteries.

## 4. Materials and Methods

### 4.1. Sources

Epoxidized soybean oil (ESO), lithium bis(fluorosulfonyl)imide (LiFSI; 99.9%), diethyl carbonate (DEC; ≥99.9%), ethylene carbonate (EC; ≥99%), lithium cobalt(III) oxide (LiCoO_2_; ≥99.8%), N-methyl pyrrolidone (NMP; 99.5%), poly(vinylidene fluoride) (PVDF; average Mw~534,000), and carbon black (99.9%) were all ordered from Sigma-Aldrich (St. Louis, MO, USA), while the lithium iron(II) phosphate (LFP) power, graphite anode, and lithium were obtained from the MTI Company (Richmond, CA, USA).

### 4.2. Characterization and Measurements

The chemical structural properties of the as-synthesized polymer electrolyte were analyzed by Fourier-transform infrared spectra (FTIR) using a Nicolet iS5 (Thermo Fisher Scientific, Waltham, MA, USA) with a scanning range of 4000–650 cm^−1^. The thermal properties were recorded using a Scinco TGA-N 1000 analyzer (Seoul, Republic of Korea) in the temperature range from 30 to 600 °C in an N_2_ atmosphere at a heating rate of 10 °C/min. The differential scanning calorimetry (DSC) was conducted on a Pyris™ Diamond DSC (PerkinElmer Co., Ltd., Waltham, MA, USA) under a nitrogen atmosphere at a heating rate of 10 °C/min over a temperature range from −60 to 250 °C.

The interfacial morphology of the as-prepared electrolyte was characterized using a field-emission scanning electron microscope (FE-SEM; JSM-7610F; JEOL, Tokyo, Japan) along with energy dispersive X-ray (EDX; inCAx-sight7421; Oxford, UK) spectroscopy. The X-ray diffraction (XRD) analysis of the as-synthesized polymer electrolyte was recorded on D2 Phase (Bruker, Germany) in the angle range from 10 to 80° at a scanning rate of 1°/min at ambient temperature.

### 4.3. Electrochemical Measurements

Electrochemical impedance spectrometers (EISs) were used to measure the ionic conductivity (σ) of the as-prepared electrolyte using an IM6ex (Zahner Elektrik GmbH & Co. KG, Kronach, Germany) instrument (at a frequency range from 0.1 to 10^5^ Hz and an AC amplitude of 5 mV in an open voltage). The fabricated asymmetrical dummy cells (Figure S3 of the SM) were allowed to reach thermal equilibrium for 30 min prior to each test. The temperature ranged from 30 to 80 °C, and each cell was recorded three times. Then, the received EIS spectra were fitted through Z-view software (version 3.1; Scribner Associates Inc., Southern Pines, NC, USA). The values of σ were calculated according to Equation (1), which is as follows:

σ = L/RS(1)

where σ (S/cm) is the ionic conductivity, R (Ω) is the bulk resistance, L (cm) represents the distance between the electrodes, and S (cm^2^) is the electrodes/electrolyte contact area.

All of the electrochemical performances of the cells were observed using Ivium-n-Stat (Ivium Technologies B.V., Eindhoven, The Netherlands). The cyclic voltammetry (CV) was examined from −1.5 to 5 V under a scan rate of 1 mV/s at 25 °C. The electrochemical anodic stability was determined by linear sweep voltammetry (LSV). CR2032-type coin cells were assembled with a stainless-steel (SS) disc as the working electrode and metallic Li as both the counter and reference electrode in the potential range from 0 to 6 V at a scan rate of 0.1 mV/s. The discharge–charge tests and cycling performances of the CR2032-type coin cells based on a graphite/C-GPEs/LFP configuration were carried out at 0.1, 0.2, and 0.3 C, respectively, under the operational voltage of 2.0 to 4.2 V and 25 °C. The C rate was defined based on the LFP cathode active material. For comparison, the half-cell of Li/C-GPE-2/LFP was assembled and examined at 0.1, 0.2, and 0.3 C, respectively, under a cell voltage of 2.5–4.2 V and 25 °C. All CR2032-type coin cells were assembled in an argon-filled glovebox (H_2_O and O_2_ < 0.1 ppm).

### 4.4. In Situ-Polymerized Cross-Linked Gel Polymer Electrolytes (C-GPEs)

The in situ polymeric electrolyte was prepared using ESO, LiFSI, and EC/DEC solvent. The digital pictures of the in situ-formed C-GPEs can be seen in Figure S4 and Video S1 of the SM for the as-prepared C-GPE-2. According to the polymerization tests, 2 M LiFSI in the electrolyte was applied in this work. First, 2 M LiFSI was added to the solution of ESO and EC/DEC (10% by *v*/*v*); the ratio in the mixture of EC/DEC is 5:5 by volume. After that, the obtained homogenous solution was placed at 40 °C in an Ar-filled glovebox for 24 h. Finally, the received electrolyte was named C-GPE-1. Similarly, based on the volume fraction of the solvent, the electrolytes with 20% and 30% solvent were denoted as C-GPE-2 and C-GPE-3, respectively. The detailed composition of the as-prepared CPEs can be seen in Table S2 in the SM. The asymmetric dummy cell was fabricated following our previous work [17] and was employed to evaluate the ionic conductivity of the as-prepared C-GPEs.

### 4.5. Preparation of LFP Cathode

The LFP cathode was composed of LFP, PVDF, and Super P at a ratio of (85:5:10)% by weight, respectively. NMP was added to the above mixture to form a homogenous slurry. Then, the slurry was coated on Al foil and dried in a vacuum oven at 120 °C for 12 h. Finally, the LFP cathode was cut into 14 mm discs and stored in a glove box before use. The loading mass of the LFP cathode was about 6 mg/cm^2^.

## Data Availability

Not applicable.

## Supplementary Materials

The following supporting information can be downloaded at https://www.mdpi.com/article/10.3390/gels9050384/s1, Figure S1: Nyquist plots of as-prepared C-GPE-1 (a), C-GPE-2 (b), and C-GPE-3 (c), and a corresponding equivalent circuit (d); Figure S2: CV profiles of C-GPE-4; Figure S3: Schematic illustration of an asymmetric dummy cell; Figure S4: Digital photos of in situ polymeric C-GPEs with varying concentrations of LiFSI; Table S1: Comparison of electrochemical properties of the polymer electrolyte with epoxy groups from reported works; Table S2: Detailed composition of the as-prepared CPEs; Figure S5: (a,b) Selected area of graphite anode of C-GPE-2 electrolyte-based LIB (inset shows the wt% of the elements C, N, O, F, and S), and (b–g) EDS elemental mapping of C, N, O, F, and S, respectively; Video S1: As-prepared C-GPE-2 after 24 h. [4,18,44,46,47,48].
