# Supplementary material for: Cross-Linked Gel Polymer Electrolyte Based on Multiple Epoxy Groups Enabling Conductivity and High Performance of Li-Ion Batteries
Source: Gels. 2023 May 5;9(5):384. doi: 10.3390/gels9050384 (PMC10217423; doi:10.3390/gels9050384)
Supplement: Supplementary file 1 [file gels-09-00384-s001.zip › Supplementary Materials.pdf]

Supplementary materials for

Crosslinked gel polymer electrolyte based on multiply-epoxy groups enable conductivity and electrochemical performance for Li-Ion Batteries

Wei Zhang [1,a], Wansu Bae[1,a], Lei Jin[1], Sungjun Park[1], Minhyuk Jeon[1], Whangi Kim[1] and Hohyoun Jang [1,*]

[1]Department of Applied Chemistry, Konkuk University, Chungju-si 27478, Republic of Korea

[a] these authors contributed equally to this work.

*Correspondence: 200417450@kku.ac.kr (H. Jang).

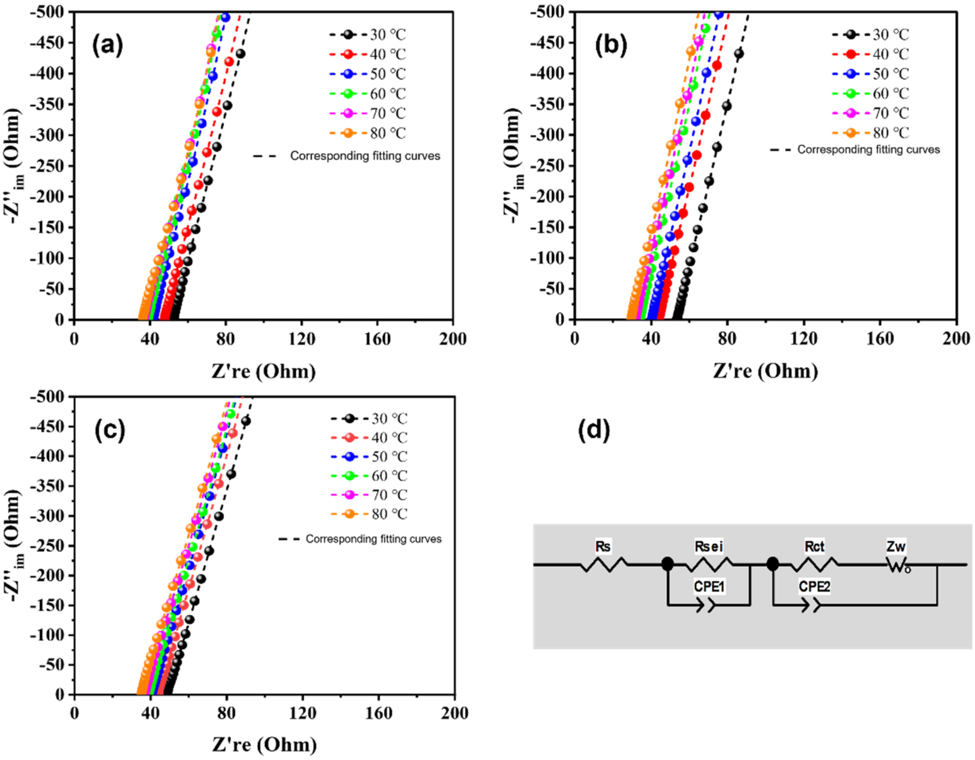

Figure S1. Nyquist plots of as-prepared C-GPE-1(a), C-GPE-2(b), and C-GPE-3(c), and corresponding an equivalent circuit(d).

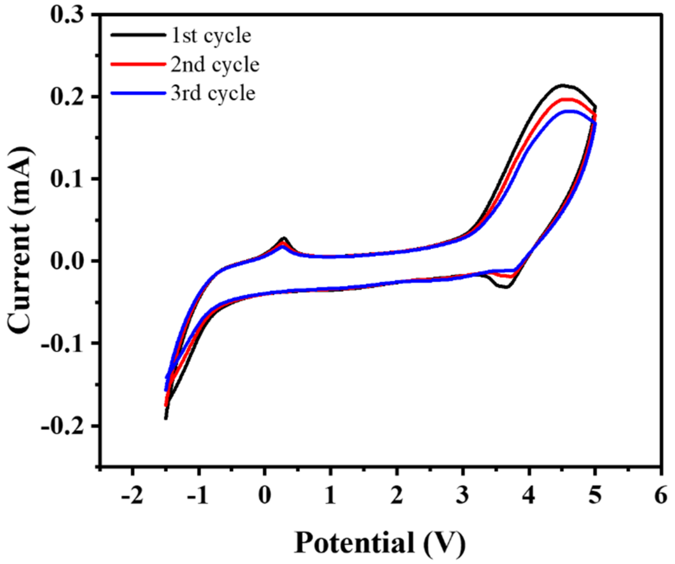

Figure S2. CV profiles of as-prepared C-GPE-4.

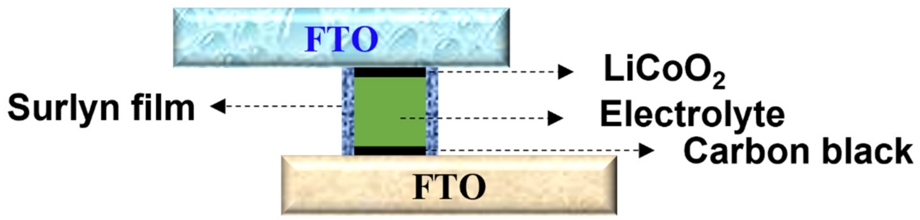

Figure S3. The schematic illustration of an asymmetric dummy cell.

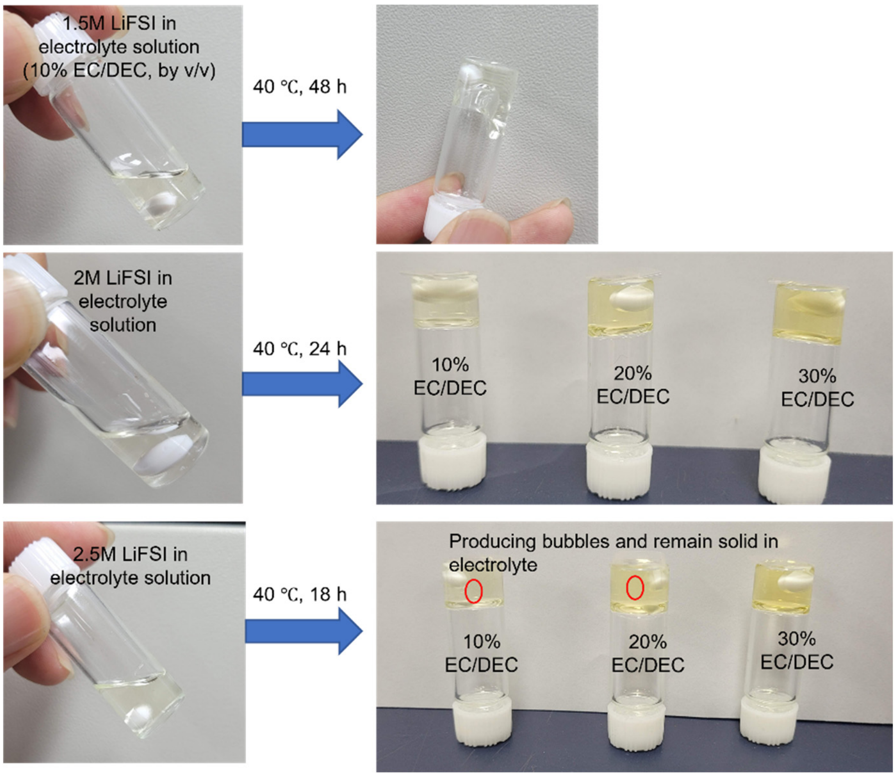

Figure S4. The digital photos of in-situ polymeric C-GPEs with varies concentrated LiFSI.

Table S1. Comparison of electrochemical properties of polymer electrolyte with epoxy group from reported works.

Electrolyte components	Ionic conductivity (S/cm at 30 ℃/ RT [a])	LSV (V, Li/Li^+)	Battery performances	Ref.
TMPEG, NPEG, LiTFSI	1.10×10^{-4}	5.4	LFP, 144.8 mAh/g, 0.2C	[1]
PEE, LiTFSI, LiDFOB	2.36×10^{-3}	4.5	NMC622, 160 mAh/g, 0.5C	[2]
NGDE, LiFSI	1.57×10^{-3}	4.0	LFP, 131 mAh/g, 0.2C	[3]
PEGDGE, LiTFSI, $LiBF_4$	1.10×10^{-4}	5.5	LFP, 160.2 mAh/g, 0.05C	[4]
GMA, OE, $LiClO_4$	HPGO-70: 2.26×10^{-6}	4.0-4.5	Li, 99 mAh/g, 0.1C	[5]
	PGO-70: 2.58×10^{-7}			
PGTE, LiDFOB	4.16×10^{-4} at 60 ℃	5.0	LFP, 135.2 mAh/g, 0.2C	[6]
C-GPE-2, LiFSI	0.23×10^{-3}	5.19	LFP, 161.3 mAh/g, 0.1C	**This work**

Note: 1) [a] Room temperature (RT)

2) $LiFePO_4$ (LFP), Trimethylolpropane triglycidyl ether (TMPEG), Poly (ethylene glycol) diamine (NPEG), Lithium bis(trifluoromethane)sulfonilimide (LiTFSI), Pentaerythritol glycidyl ether (PEE), $LiNi_{0.6}Mn_{0.2}Co_{0.2}O_2$ (NMC622), Lithium difluoro(oxalate)borate (LiDFOB), Neopentyl glycol diglycidyl ether (NGDE), Poly(ethylene glycol) diglycidyl ether (PEGDGE), Lithium tetrafluoroborate ($LiBF_4$), Glycidyl methacrylate (GMA), Oligo (ethylene oxide) methyl ether methacrylate (OE), Lithium perchlorate ($LiClO_4$), Polymerized glycerin triglycidyl ether (PGTE)

Table S2. The composition of the as-prepared crosslinked-gel polymer electrolyte in this work

Crosslinked-Gel Polymer electrolytes (C-GPEs) names	ESO (mL)	LiFSI (g)	EC/DEC (mL)
**C-GPE-1**	2	0.748	0.2
**C-GPE-2**			0.4
**C-GPE-3**			0.6

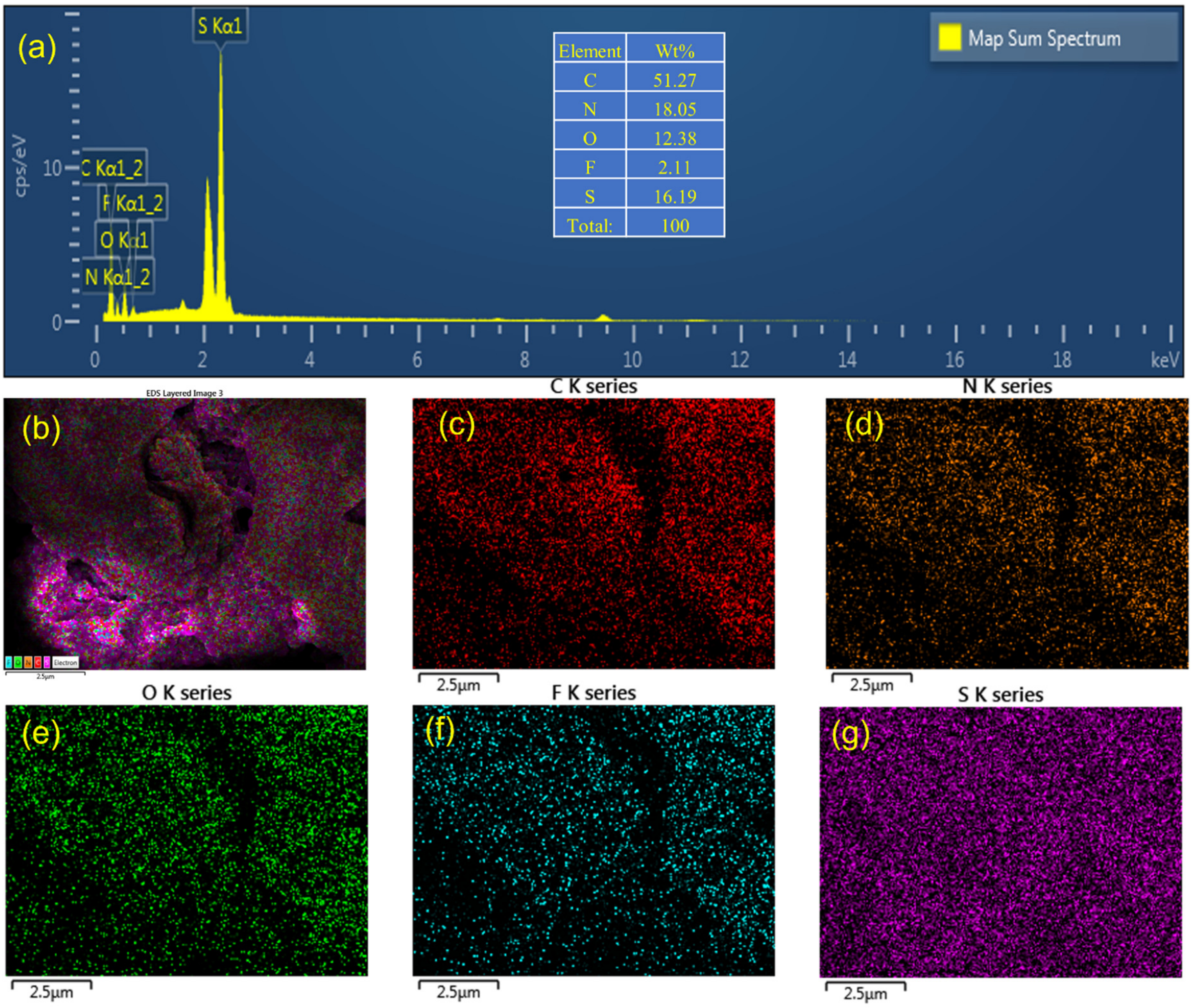

Figure S5: (a-b) Selected area of graphite anode of C-GPE-2 electrolyte based LIB (inset shows the wt% of elemental C, N, O, F, and S), and (b-g) EDS elemental mapping of C, N, O, F, and S, respectively. The graphite anode was recovered after 300 CD cycles.

Reference

1. Chen, B.; Xu, Q.; Huang, Z.; Zhao, Y.; Chen, S.; Xu, X. One-pot preparation of new copolymer electrolytes with tunable network structure for all-solid-state lithium battery. J. Power Sources 2016, 331, 322-331. doi:10.1016/j.jpowsour.2016.09.063.
2. Zhu, J.; Zhang, J.; Zhao, R.; Zhao, Y.; Liu, J.; Xu, N.; Wan, X.; Li, C.; Ma, Y.; Zhang, H.; Chen, Y. In situ 3D crosslinked gel polymer electrolyte for ultra-long cycling, high-voltage, and high-safety lithium metal batteries. Energy Storage Materials 2023, 57, 92-101. doi:10.1016/j.ensm.2023.02.012.
3. Zhang, W.; Ryu, T.; Yoon, S.; Jin, L.; Jang, G.; Bae, W.; Kim, W.; Ahmed, F.; Jang, H. Synthesis and Characterization of Gel Polymer Electrolyte Based on Epoxy Group via Cationic Ring-Open Polymerization for Lithium-Ion Battery. Membranes 2022, 12, 439. doi:10.3390/membranes12040439.
4. Nair, J.R.; Shaji, I.; Ehteshami, N.; Thum, A.; Diddens, D.; Heuer, A.; Winter, M. Solid Polymer Electrolytes for Lithium Metal Battery via Thermally Induced Cationic Ring-Opening Polymerization (CROP) with an Insight into the Reaction Mechanism. Chem. Mater. 2019, 31, 3118-3133. doi:10.1021/acs.chemmater.8b04172.
5. Yao, W.; Zhang, Q.; Qi, F.; Zhang, J.; Liu, K.; Li, J.; Chen, W.; Du, Y.; Jin, Y.; Liang, Y.; Liu N. Epoxy containing solid polymer electrolyte for lithium ion battery. Electrochim. Acta 2019, 318, 302-313. doi:10.1016/j.electacta.2019.06.069.
6. Ma, Y.; Sun, Q.; Wang, S.; Zhou, Y.; Song, D.; Zhang, H.; Shi, X.; Zhang, L. Li salt initiated in-situ polymerized solid polymer electrolyte: new insights via in-situ electrochemical impedance spectroscopy. Chem. Eng. J. 2022, 429, 132483. doi:10.1016/j.cej.2021.132483.
